# Preoperative peripheral inflammatory markers are predictors of postoperative central diabetes insipidus in craniopharyngioma patients: a retrospective study

**DOI:** 10.1186/s12885-024-12324-4

**Published:** 2024-05-08

**Authors:** Jing Wang, Guanghui Wang, Lidong Cheng, Hongtao Zhu, Junwen Wang, Xinmin Ding, Hongquan Niu, Kai Zhao, Kai Shu

**Affiliations:** 1 Department of Neurosurgery, Tongji Hospital, Tongji Medical College, Huazhong University of Science and Technology, Wuhan, 430030 China; 2 Department of Neurosurgery, Shanxi Bethune Hospital, Shanxi Academy of Medical Sciences, Tongji Shanxi Hospital, Third Hospital of Shanxi Medical University, Taiyuan, 030032 China

**Keywords:** Craniopharyngioma, Diabetes insipidus, Inflammatory marker, Lymphocyte, Neutrophil-to-lymphocyte ratio

## Abstract

**Background:**

Postoperative central diabetes insipidus (CDI) is commonly observed in craniopharyngioma (CP) patients, and the inflammatory response plays an important role in CPs. We aimed to evaluate the predictive value of preoperative peripheral inflammatory markers and their combinations regarding CDI occurrence in CPs.

**Methods:**

The clinical data including preoperative peripheral inflammatory markers of 208 CP patients who underwent surgical treatment were retrospectively collected and analyzed. The preoperative peripheral white blood cells (WBC), neutrophils, lymphocytes, monocytes, platelet (PLT), neutrophil-to-lymphocyte ratio (NLR), derived-NLR (dNLR), monocyte-to-lymphocyte ratio (MLR) and PLT-to-lymphocyte ratio (PLR) were assessed in total 208 CP patients and different age and surgical approach CP patient subgroups. Their predictive values were evaluated by the receiver operator characteristic curve analysis.

**Results:**

Preoperative peripheral WBC, neutrophils, NLR, dNLR, MLR, and PLR were positively correlated and lymphocyte was negatively associated with postoperative CDI occurrence in CP patients, especially when WBC ≥ 6.66 × 10^9^/L or lymphocyte ≤ 1.86 × 10^9^/L. Meanwhile, multiple logistic regression analysis showed that WBC > 6.39 × 10^9^/L in the > 18 yrs age patients, WBC > 6.88 × 10^9^/L or lymphocytes ≤ 1.85 × 10^9^/L in the transcranial approach patients were closely associated with the elevated incidence of postoperative CDI. Furthermore, the area under the curve obtained from the receiver operator characteristic curve analysis showed that the best predictors of inflammatory markers were the NLR in total CP patients, the MLR in the ≤ 18 yrs age group and the transsphenoidal group, the NLR in the > 18 yrs age group and the dNLR in the transcranial group. Notably, the combination index NLR + dNLR demonstrated the most valuable predictor in all groups.

**Conclusions:**

Preoperative peripheral inflammatory markers, especially WBC, lymphocytes and NLR + dNLR, are promising predictors of postoperative CDI in CPs.

**Supplementary Information:**

The online version contains supplementary material available at 10.1186/s12885-024-12324-4.

## Introduction

Craniopharyngioma (CP) is a common benign tumor arising from squamous cell nests in the primitive Rathke’s pouch in the central nervous system. The point prevalence of CPs is approximately 0.5–2.5/1,000,000 in the population [1]. Accompanied by increased knowledge of CPs and improved endoscopic surgery and radiation therapy, the surgical outcomes for CPs have significantly improved [2, 3]. However, the postoperative process in each patient is different due to various postoperative complications, including central diabetes insipidus (CDI) and multiple pituitary hormone deficiencies [2].

CDI is the most common complication in CPs after surgery mainly resulting from the impairment of hypothalamic posterior pituitary function [1]. Briefly, postoperative CDI is characterized as a triphasic response of urine volume. The prevalence of postoperative CDI in CPs has been reported elsewhere to be up to 90% [4]. Although CDI has been well studied for decades, the management of this entity remains controversial. Numerous factors for the prediction of postoperative CDI occurrence have been identified in previous publications, such as patient age, tumor histopathological type, tumor volume, and cerebrospinal fluid leakage [5, 6].

A cerebral inflammatory response is often observed and displays a close relationship with tumor prognosis in multiple tumors, including CPs [7, 8]. Increasing studies have demonstrated that inflammatory cytokines, including IL-6, IL-8, CXCL1, etc., are closely related to CPs development [9, 10]. Although these inflammatory factors have shown predictive value, medical expenses have limited their clinical application. Thus, finding a simpler, readily available, inexpensive method is urgent. Numerous studies have demonstrated that peripheral inflammatory markers and their combinations can be used for monitoring nonspecific inflammatory responses in various pathological situations [11–13], as well as inflammation monitoring, differential diagnosis, and prognosis prediction of CPs [7, 13–15]. However, there remains a lack of comprehensive investigations into whether there are correlations between preoperative peripheral inflammatory markers and postoperative CDI in CPs, which is the purpose of this study.

## Methods

### Patients

The clinical data of 267 patients diagnosed with CPs who underwent surgical treatment in the neurosurgery department of Tongji Hospital, Tongji Medical College, Huazhong University of Science and Technology, from January 2016 to October 2021 were retrospectively collected and analyzed. The exclusion criteria were as follows: (1) patients with preoperative CDI; (2) patients who underwent radiotherapy or surgical treatment before; (3) patients with an infectious disease or severe organ dysfunction, such as failure of the lung, heart, liver and kidney; (4) patients with incomplete clinical data; and (5) patients taking diuretics or anti-inflammatory reagents postoperatively.

### Data collection and study design

Clinical variables, including sex, age, laboratory examinations and surgical approach, were recorded directly from the medical records. The location of tumors was analysed from magnetic resonance images (MRI) performed by two independent experienced senior neurosurgeons and classified into Q-type, S-type and T-type according to the criteria described in a previous report [16]. The volumes of tumors were measured with preoperative MRI and the extent of tumor resection was evaluated by postoperative MRI and described as gross total resection (GTR, more than 95% of tumor resected) or subtotal resection (STR, the residual tumor volume was less than 20%) [17, 18]. The blood specimens for laboratory examination in our current study were collected from each patient at admission, daily during the first week after the operation and every 2–3 days during the subsequent weeks postoperatively. The preoperative pituitary functions were evaluated with blood tests and patients with hypopituitarism were given preoperative steroid replacement (PSR) treatment until the cortisone supplementation was adequate, and then the surgery was performed. The counts of white blood cells (WBC), neutrophils, lymphocytes, monocytes and platelet (PLT) were directly collected from the peripheral blood tests and the combined inflammatory markers, including NLR (neutrophil-to-lymphocyte ratio; NLR = neutrophils/lymphocytes), dNLR [derived-NLR; dNLR = (WBC-neutrophils)/lymphocytes], MLR (monocyte-to-lymphocyte ratio; MLR = monocytes/lymphocytes) and PLR (platelet -to-lymphocyte ratio; PLR = platelet /lymphocytes), were calculated. CDI was identified by two independently experienced neurosurgeons during treatments for CPs in the first week postoperatively, in terms of the diagnosis described previously [19]. In general, the laboratory tests used for monitoring CDI include: (1) increased urine output (more than 300 ml for 3 consecutive hours); (2) elevated serum sodium concentration (higher than 145 mmol/l); and (3) reduced urine specific gravity (less than 1.005).

### Statistical analysis

All statistical analyses were performed using IBM SPSS statistics software, version 23.0. For the normally and abnormally distributed data, continuous variables were described as the mean ± standard deviation and median (interquartile range, IQR) [M (P25, P75)], respectively. Differences between the two independent groups were compared by Student’s independent t-test and the Mann-Whitney U test. Qualitative data were summarized as counts and percentages and were analyzed using the Chi-square tests or Fisher’s exact tests (expected count ≤ 5). We used multiple logistic regression to identify independent predictors of preoperative peripheral inflammatory markers for postoperative CDI of CPs patients. Meanwhile, to evaluate the predictive value of inflammatory markers on early postoperative CDI, we drew a receiver operator characteristic (ROC) curve. The area under the curve (AUC) was automatically calculated. *p* < 0.05 was considered statistically significant.

## Results

### Patients’ characteristics, peripheral inflammatory markers and CDI data

Of the 208 patients, postoperative CDI was identified in 93 patients (44.7%). Numerous clinical data regarding age, age groups, sex, surgical approach, tumor volume, preoperative pituitary functions, extent of tumor resection and tumor location displayed no significant differences between the CDI and non-CDI groups. The preoperative WBC and neutrophil counts in patients with postoperative CDI were significantly higher than those in patients without CDI (*p* < 0.001 and *p* = 0.011, respectively), while the lymphocyte count in patients with CDI was lower (*p* = 0.008). The monocytes and PLT did not show a significant difference between the CDI and non-CDI groups. In addition, the combined inflammatory markers NLR, MLR, dNLR and PLR displayed higher levels in CPs with CDI than in CPs without CDI (all *p* < 0.05) (Table 1).

**Table 1:** Clinical analysis of postoperative CDI in craniopharyngioma patients

Variants	Total (*n* = 208)	CDI (*n* = 93)	Non-CDI (*n* = 115)	*p*-value
Age (yrs)	41.0 (22.0, 51.8)	39.0 (23.5, 51.0)	41.0 (22.0, 52.0)	0.650
Age groups				0.849
≤ 18 yrs	46	20 (43.5%)	26 (56.5%)	
>18 yrs	162	73 (45.1%)	89 (54.9%)	
Gender				0.488
Male	124	53 (42.7%)	71 (57.3%)	
Female	84	40 (47.6%)	44 (52.4%)	
Surgical approach				0.466
Transcranial	189	83 (43.9%)	106 (56.1%)	
Transsphenoidal	19	10 (52.6%)	9 (47.4%)	
Extent of tumor resection				0.476
GTR	191	84 (44.0%)	107 (56.0%)	
STR	17	9 (52.9%)	8 (47.1%)	
Tumor location				0.444
Q-type	87	39 (44.8%)	48 (55.2%)	
S-type	98	41 (41.8%)	57 (58.2%)	
T-type	23	13 (56.5%)	10 (43.5%)	
Pituitary function				0.494
hypopituitarism with PSR	18	9 (50.0%)	9 (50.0%)	
None	190	84 (44.2%)	106 (55.8%)	
Tumor volume				0.234
< 5 cm^3^	133	44 (33.1%)	89 (66.9%)	
≥ 5 cm^3^	75	31 (41.3%)	44 (58.7%)	
Inflammatory marker				
WBC (×10^9^/L)	6.66 (5.74, 8.90)	7.53 (6.03, 9.79)	6.22 (5.28, 8.13)	<0.001***
Neutrophil (×10^9^/L)	3.73 (2.88, 5.51)	4.06 (3.14, 5.93)	3.55 (2.65, 4.99)	0.011*
Lymphocyte(×10^9^/L)	1.86 (1.46, 2.38)	1.71 (1.37, 2.18)	1.94 (1.57, 2.54)	0.008**
Monocyte (×10^9^/L)	0.45 (0.35, 0.55)	0.45 (0.36, 0.55)	0.44 (0.35, 0.57)	0.888
PLT (×10^9^/L)	215.0 (172.3, 266.5)	211.0 (170.5, 260.0)	223.0 (174.0, 267.0)	0.353
NLR	1.99 (1.42, 3.44)	2.30 (1.54, 4.96)	1.79 (1.28, 2.69)	<0.001***
MLR	0.23 (0.17, 0.31)	0.25 (0.18, 0.33)	0.22 (0.16, 0.30)	0.013*
dNLR	1.34 (1.24, 1.62)	1.40 (1.27, 2.96)	1.32 (1.22, 1.44)	0.001**
PLR	109.7 (87.3, 155.0)	118.1 (90.8, 164.0)	105.6 (84.9, 139.1)	0.029*

*p<0.05, **p<0.01, and ***p<0.001. CDI: central diabetes insipidus; PSR: preoperative steroid replacement; GTR: gross total resection; STR: subtotal resection; WBC: white blood cells; PLT: platelet; NLR: neutrophil-to-lymphocyte ratio; MLR: monocyte-to-lymphocyte ratio; dNLR: derived NLR; PLR: platelet-to-lymphocyte ratio

Multiple logistic regression analysis showed that the incidence of CDI was increased when preoperative WBC was higher than 6.66 × 10^9^/L (*p* = 0.002) or lymphocyte was lower than 1.86 × 10^9^/L (*p* = 0.022) (Table 2).

**Table 2:** The multivariate logistic regression analysis of risk factors for CDI occurrence

Variants	B	Wald	*p*-value	OR	95%CI
WBC ≥ 6.66 (×10^9^/L)	-1.233	9.274	0.002**	0.291	0.132–0.644
Neutrophil ≥ 3.73 (×10^9^/L)	0.277	0.357	0.550	1.319	0.532–3.275
Lymphocyte ≤ 1.86 (×10^9^/L)	0.896	5.260	0.022*	2.450	1.139–5.268
NLR ≥ 1.99	0.046	0.010	0.918	1.047	0.436–2.514
MLR ≥ 0.23	-0.186	0.245	0.620	0.830	0.397–1.734
dNLR ≥ 1.34	0.253	0.452	0.501	1.287	0.616–2.688
PLR ≥ 109.7	-0.330	1.104	0.293	0.719	0.389–1.330

*p<0.05 and **p<0.01. CDI: central diabetes insipidus; WBC: white blood cells; NLR: neutrophil-to-lymphocyte ratio; MLR: monocyte-to-lymphocyte ratio; dNLR: derived NLR; PLR: platelet-to-lymphocyte ratio

In addition, although there was no significant difference, compared with patients with hypopituitarism and received PSR, patients with normal pituitary function had a tendency of higher levels of preoperative inflammatory markers including WBC, neutrophil, monocyte, PLT, NLR, MLR and PLR (Additional Table 1).

### Clinical analysis in different subgroups

To evaluate the impact of preoperative inflammatory markers on postoperative CDI in young and adult patients, we divided participants into two age subgroups. As shown in Table 3, the WBC, neutrophils and NLR displayed significant differences between the CDI and non-CDI in the > 18 yrs age group (WBC: *p* = 0.001, neutrophils: *p* = 0.024 and NLR: *p* = 0.003), but not in ≤ 18 yrs age group (all *p* > 0.05). The MLR was elevated in the ≤ 18 yrs age group with CDI compared with those without CDI (*p* = 0.007). However, the MLR was not evaluated differently in the > 18 yrs age group with and without postoperative CDI. Moreover, the dNLR was closely related to CDI occurrence in both ≤ 18 and > 18 yrs age groups (*p* = 0.014 and *p* = 0.011, respectively).

**Table 3:** Clinical analysis of postoperative CDI in young and adult craniopharyngioma patients

Variants	Age ≤ 18 yrs (*n* = 46)		*p*-value	Age > 18 yrs (*n* = 162)		*p*-value
	CDI (*n* = 20)	Non-CDI (*n* = 26)		CDI (*n* = 73)	Non-CDI (*n* = 89)	
WBC (×10^9^/L)	8.49 ± 2.68	7.62 ± 2.10	0.225	7.36 (5.96, 9.88)	6.14 (5.26, 7.84)	0.001**
Neutrophil (×10^9^/L)	4.53 (3.14, 6.55)	3.50 (2.62, 5.48)	0.227	3.96 (3.12, 5.88)	3.62 (2.75, 4.51)	0.024*
Lymphocyte (×10^9^/L)	1.96 (1.33, 2.65)	2.31 (1.93, 3.05)	0.061	1.71 (1.39, 2.16)	1.87 (1.48, 2.37)	0.0952
Monocyte (×10^9^/L)	0.52 (0.41, 0.64)	0.43 (0.39, 0.60)	0.263	0.43 ± 0.14	0.45 ± 0.17	0.480
PLT (×10^9^/L)	285.0 ± 83.2	309.0 ± 92.5	0.368	201.5 ± 54.5	208.3 ± 59.7	0.457
NLR	2.12 (1.37, 5.58)	1.42 (0.89, 2.25)	0.051	2.54 (1.63, 4.96)	1.89 (1.43, 2.72)	0.003**
MLR	0.29 (0.19, 0.41)	0.20 (0.13, 0.22)	0.007	0.25 (0.18, 0.32)	0.23 (0.16, 0.31)	0.240
dNLR	1.45 (1.26, 2.88)	1.27 (1.21, 1.41)	0.014	1.35 (1.27, 3.17)	1.34 (1.22, 1.45)	0.011*
PLR	143.5 (109.6, 213.8)	119.1 (86.8, 161.1)	0.126	111.9 (88.0, 151.7)	103.1 (83.9, 133.9)	0.108

*p<0.05 and **p<0.01. CDI: central diabetes insipidus; WBC: white blood cells; PLT: platelet; NLR: neutrophil-to-lymphocyte ratio; MLR: monocyte-to-lymphocyte ratio; dNLR: derived NLR; PLR: platelet-to-lymphocyte ratio

Next, we further explored the preoperative inflammatory markers in patients who underwent surgery using different surgical approaches (Table 4). Of 189 patients undergoing the transcranial approach, the WBC, neutrophils, NLR and dNLR were predominantly higher in the CDI group than in the non-CDI group (WBC: *p* = 0.001, neutrophils: *p* = 0.030, NLR: *p* = 0.004 and dNLR: *p* = 0.003), while the lymphocytes was lower (*p* = 0.023). In addition, the monocyte, MLR and dNLR were associated with CDI prevalence in the transsphenoidal approach patients (monocytes: *p* = 0.017, MLR: *p* = 0.011 and dNLR: *p* = 0.022).

**Table 4:** Clinical analysis of postoperative CDI in craniopharyngioma patients with different surgical approaches

Variants	Transcranial (*n* = 189)		*p*-value	Transsphenoidal (*n* = 19)		*p*-value
	CDI (*n* = 83)	Non-CDI (*n* = 106)		CDI (*n* = 10)	Non-CDI (*n* = 9)	
WBC (×10^9^/L)	7.53 (6.10, 9.85)	6.39 (5.58, 8.35)	0.001**	7.52 (5.33, 8.99)	5.23 (4.69, 6.21)	0.050
Neutrophil (×10^9^/L)	4.01 (3.22, 5.95)	3.63 (2.84, 5.09)	0.030*	4.88 ± 3.03	2.83 ± 1.01	0.071
Lymphocyte (×10^9^/L)	1.74 (1.38, 2.19)	1.92 (1.50, 2.55)	0.023*	1.67 ± 0.72	2.14 ± 0.38	0.099
Monocyte (×10^9^/L)	0.46 (0.35, 0.55)	0.45 (0.36, 0.58)	0.720	0.47 ± 0.11	0.34 ± 0.11	0.017*
PLT (×10^9^/L)	211.0 (168.0, 263.0)	227.0 (174.8, 268.0)	0.337	208.7 ± 62.3	210.8 ± 73.9	0.948
NLR	2.28 (1.53, 4.48)	1.84 (1.33, 2.79)	0.004**	4.04 (1.63, 5.22)	1.39 (0.72, 2.01)	0.050
MLR	0.25 (0.18, 0.32)	0.22 (0.16, 0.30)	0.077	0.26 (0.19, 0.50)	0.13 (0.12, 0.23)	0.011*
dNLR	1.40 (1.26, 3.01)	1.33 (1.23, 1.44)	0.003**	1.41 (1.29, 2.20)	1.18 (1.16, 1.37)	0.022*
PLR	118.1 (91.7, 162.4)	105.2 (85.3, 139.3)	0.055	122.8 (82.2, 200.8)	109.3 (65.2, 130.8)	0.288

*p<0.05 and **p<0.01. CDI: central diabetes insipidus; WBC: white blood cells; PLT: platelet; NLR: neutrophil-to-lymphocyte ratio; MLR: monocyte-to-lymphocyte ratio; dNLR: derived NLR; PLR: platelet-to-lymphocyte ratio

Furthermore, multiple logistic regression analysis showed that the incidence of CDI was increased when preoperative WBC was higher than 6.39 × 10^9^/L (*p* = 0.035) in > 18 yrs age group, WBC was higher than 6.88 × 10^9^/L (*p* = 0.022) or lymphocyte was lower than 1.85 × 10^9^/L (*p* = 0.029) in transcranial group, respectively (Table 5).

**Table 5:** Multivariate logistic regression analysis of risk factors for CDI occurrence

Variants	B	Wald	*p*-value	OR	95%CI
**Age** ≤ **18 yrs (***n* **=** **46)**					
MLR ≥ 0.21	-1.455	2.390	0.122	0.233	0.037–1.476
dNLR ≥ 1.31	-0.038	0.002	0.967	0.962	0.153–6.058
**Age** > **18 yrs (***n* **=** **162)**					
WBC ≥ 6.39 (×10^9^/L)	-0.932	4.431	0.035*	0.394	0.165–0.938
Neutrophil ≥ 3.72 (×10^9^/L)	0.611	1.343	0.246	1.842	0.656–5.177
NLR ≥ 2.10	-0.779	3.139	0.076	0.459	0.194–1.086
dNLR ≥ 1.35	0.205	0.341	0.559	1.228	0.616–2.445
**Transcranial (***n* **=** **189)**					
WBC ≥ 6.88(×10^9^/L)	-0.970	5.235	0.022*	0.379	0.165–0.870
Neutrophil ≥ 3.73(×10^9^/L)	0.185	0.150	0.699	1.203	0.472–3.065
Lymphocyte ≤ 1.85(×10^9^/L)	0.821	4.748	0.029*	2.272	1.086–4.755
NLR ≥ 1.99	-0.032	0.005	0.943	0.968	0.404–2.322
dNLR ≥ 1.35	-0.672	0.488	0.485	0.511	0.078–3.365
**Transsphenoidal (***n* **=** **19)**					
Monocyte ≥ 0.40(×10^9^/L)	-1.393	1.049	0.306	0.248	0.017–3.571
MLR ≥ 0.22	0.163	0.012	0.913	1.177	0.064–21.651
dNLR ≥ 1.31	-0.585	0.239	0.625	0.557	0.053–5.818

*p<0.05. CDI: central diabetes insipidus; MLR: monocyte-to-lymphocyte ratio; dNLR: derived NLR; WBC: white blood cells; NLR: neutrophil-to-lymphocyte ratio

### ROC curve analysis and predictive values

**Fig. 1:**
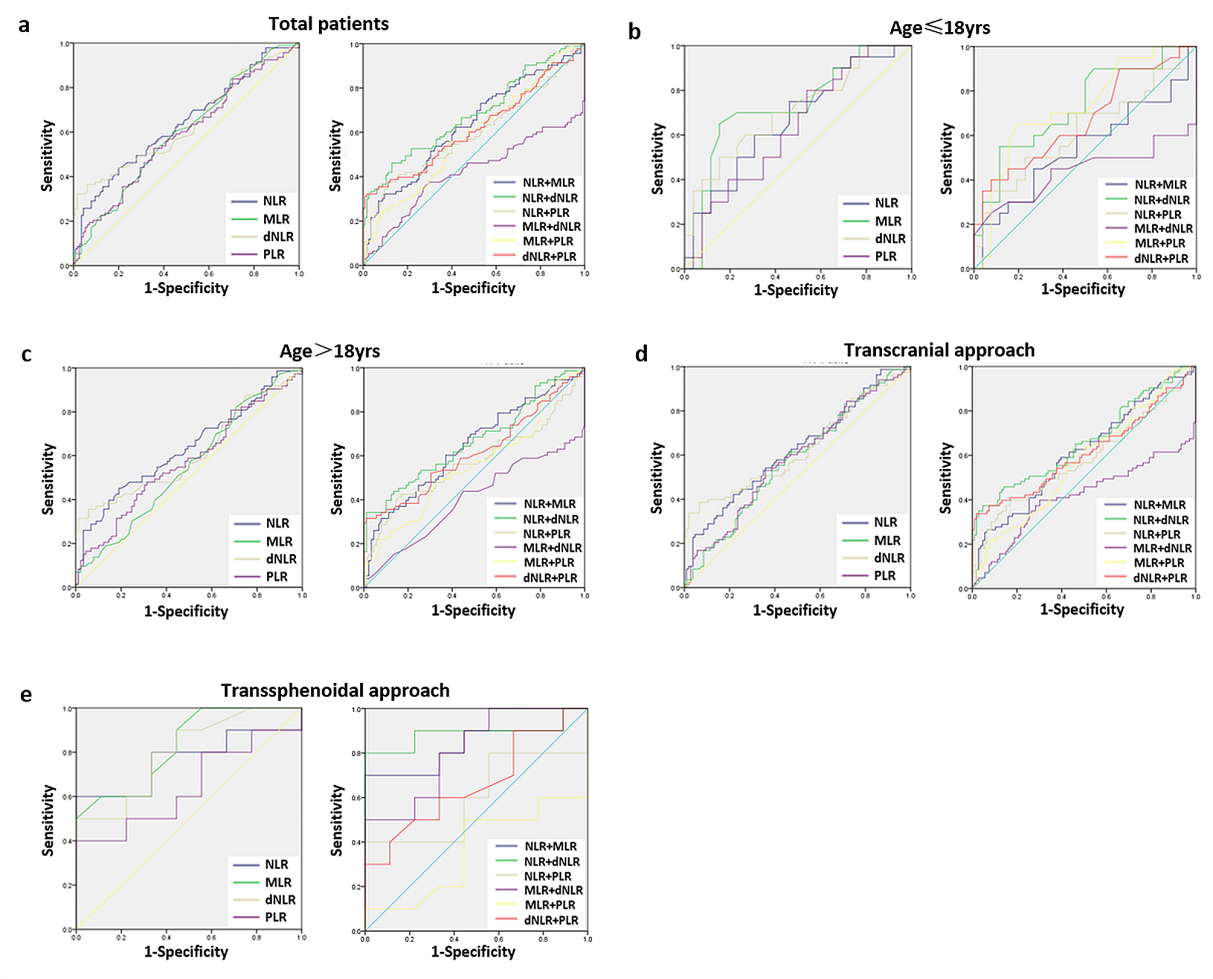
The predictive value of the preoperative peripheral inflammatory markers. The predictive value of NLR, MLR, dNLR, PLR and their combinations in 208 CP patients (**a**), ≤ 18 yrs age group (**b**), > 18 yrs age group (**c**), transcranial approach group (**d**), and transsphenoidal approach group (**e**)

The corresponding ROC curves and AUC are shown in Table 6; Fig. 1. Among the 208 CP patients, the AUCs were 0.641 (0.565–0.717) for NLR, which demonstrated the highest accuracy in predicting CDI occurrence. The evaluation of paired combinations of these inflammatory makers indicated that NLR + dNLR was the best predictor with an AUC of 0.681 (0.607–0.755) (Fig. 1a). Further investigation revealed that the best accuracy for predicting CDI was obtained with MLR [AUC: 0.729 (0.580–0.878)] and NLR + dNLR [AUC: 0.731 (0.579–0.883)] in the ≤ 18 yrs age group (Fig. 1b); NLR [AUC: 0.635 (0.547–0.722)] and NLR + dNLR [AUC: 0.667 (0.582–0.753)] in the > 18 yrs age group (Fig. 1c); dNLR [AUC: 0.628 (0.545–0.711)] and NLR + dNLR [AUC: 0.661 (0.581–0.742)] in the transcranial approach group (Fig. 1d); and MLR [AUC: 0.828 (0.643-1.000)] and NLR + dNLR [AUC: 0.889 (0.716-1.000)] in the transsphenoidal approach group (Fig. 1e). Notably, among all inflammatory markers and their paired combinations, NLR + dNLR might be a discriminative parameter for predicting the prevalence of postoperative CDI.

**Table 6:** Predictive value of the preoperative inflammatory marker and their combinations

Variants	AUC (95%CI)				
	Total	Age ≤ 18yrs	Age > 18yrs	Transcranial	Transsphenoidal
NLR	0.641 (0.565–0.717)	0.669 (0.512–0.827)	0.635 (0.547–0.722)	0.622 (0.541–0.703)	0.767 (0.539–0.995)
MLR	0.598 (0.521–0.675)	0.729 (0.580–0.878)	0.552 (0.464–0.641)	0.575 (0.493–0.657)	0.828 (0.643-1.000)
dNLR	0.638 (0.560–0.715)	0.709 (0.556–0.861)	0.614 (0.524–0.704)	0.628 (0.545–0.711)	0.800 (0.601–0.999)
PLR	0.588 (0.510–0.666)	0.633 (0.472–0.793)	0.574 (0.484–0.663)	0.581 (0.499–0.663)	0.644 (0.388-0.900)
NLR + MLR	0.632 (0.556–0.709)	0.542 (0.367–0.718)	0.643 (0.556–0.729)	0.610 (0.529–0.692)	0.833 (0.638-1.000)
NLR + dNLR	0.681 (0.607–0.755)	0.731 (0.579–0.883)	0.667 (0.582–0.753)	0.661 (0.581–0.742)	0.889 (0.716-1.000)
NLR + PLR	0.596 (0.516–0.676)	0.622 (0.453–0.791)	0.572 (0.479–0.665)	0.581 (0.496–0.666)	0.600 (0.331–0.869)
MLR + dNLR	0.420 (0.337–0.503)	0.439 (0.248–0.630)	0.402 (0.311–0.494)	0.415 (0.327–0.504)	0.811 (0.617-1.000)
MLR + PLR	0.585 (0.507–0.663)	0.727 (0.577–0.877)	0.556 (0.466–0.647)	0.571 (0.489–0.654)	0.361 (0.103–0.620)
dNLR + PLR	0.619 (0.539–0.698)	0.671 (0.511–0.832)	0.614 (0.524–0.704)	0.613 (0.528–0.698)	0.661 (0.412–0.910)

NLR: neutrophil-to-lymphocyte ratio; MLR: monocyte-to-lymphocyte ratio; dNLR: derived NLR; PLR: platelet-to-lymphocyte ratio

## Discussion

Despite the fact that a long-term survival rate has been achieved in CP patients, the postoperative process is different in each individual, mainly resulting from multiple complications, particularly from CDI and the subsequent disturbance of water and electrolytes [19]. The incidence rate of CDI after surgery in CPs has been reported in an extensive range of 1.6–93% [5, 6]. In our study, postoperative CDI was identified in 93 CP patients (44.7%), revealing a similar incidence to that in most previous reports. Inflammatory markers have been reported to play essential roles in tumor development, tumoral calcification, patient prognosis and the prevalence of postoperative complications including hypopituitarism [7, 20, 21]. However, little is known about the impact of the inflammatory markers on postoperative CDI in CPs.

Chronic inflammation plays pivotal roles in various brain diseases, such as hydrocephalus, cerebral hemorrhage, cerebral infarction, traumatic brain injury and gliomas [8, 22, 23]. Most recently, the inflammatory response in CPs has been well studied [9, 24, 25]. To quantitatively evaluate the inflammatory response, various inflammatory markers, either in cerebrospinal fluid (CSF) or in peripheral blood, have been evaluated in previous publications. The detection of inflammatory markers in CSF could directly reflect the inflammatory response in the brain. However, the clinical application has been limited for the following reasons: (1) CSF acquisition was invasive and inconvenient; (2) the value of inflammatory markers in CSF lacked certain reference intervals; and (3) these markers in CSF could not be detected continuously. Therefore, in our current retrospective study, we explored whether preoperative peripheral inflammatory markers and their combinations were associated with postoperative CDI occurrence in CPs.

The peripheral levels of WBC, neutrophils, lymphocytes and monocytes are identified as general markers of nonspecific inflammatory responses in the central nervous system (CNS). Increasing clinical data have indicated that peripheral WBC, neutrophils and monocytes play proinflammatory roles, while lymphocytes dominantly participate in the anti-inflammatory procedures [26, 27]. For example, WBC and neutrophil counts were positively correlated with the malignancy of gliomas [28]. A higher peripheral blood WBC count and lower lymphocyte count indicated a poor prognosis in glioblastoma patients [29, 30]. Moreover, higher levels of preoperative WBC and neutrophils might be potential markers to differentially diagnose papillary CPs [7]. In our study, we observed that higher WBC (≥ 6.66 × 10^9^/L) or lower lymphocyte (≤ 1.86 × 10^9^/L) in the total CP patients (Tables 1 and 2), higher WBC (> 6.39 × 10^9^/L) in the > 18 yrs age patients, higher WBC (> 6.88 × 10^9^/L) or lower lymphocytes (≤ 1.85 × 10^9^/L) in the transcranial approach patients (Tables 3, 4 and 5) were closely associated with the elevated incidence of postoperative CDI. All of the above suggested that the inflammatory response might be closely associated with the prevalence of postoperative CDI in CPs and the WBC and lymphocytes may be the high-risk factors. Moreover, the WBC did not show differences between young patients with CDI and without CDI (Table 3). This result might have occurred because of: (1) the different clinical features of pediatric CPs; and (2) the statistical bias from the small young CP population in the current study. In addition, the dual function of monocytes has been demonstrated in various tumors [38]. However, monocyte function in CPs remains uncertain. Here, we report a relatively lower monocyte count in patients without CDI than in those with CDI after transsphenoidal surgery (Table 4). Unfortunately, multiple logistic regression analysis revealed that monocyte was insufficient for increasing the risk of postoperative CDI in patients with CPs (Table 5).

Considering the coexistence of pro-inflammation and anti-inflammation in the pathological circumstances of patients, the combined inflammatory markers NLR, dNLR, PLR and MLR have been used to evaluate the balance between pro- and anti-inflammation [20, 31]. Moreover, these combined markers were more reproducible and accurate than routine blood cell counts. Higher NLR and MLR were associated with elevated mortality, neurological deterioration and poor outcome in cerebral hemorrhage patients [22, 32]. In glioma patients, elevated NLR, PLR and MLR are reliable predictors of a poor outcome [8, 33]. A higher NLR was correlated with a poor outcome in CPs [15]. In addition, PLR played roles in predicting neurological outcomes in comparison to PLT count alone [34]. Our study found that CP patients with postoperative CDI had higher levels of combined inflammatory markers, including NLR, MLR, dNLR and PLR (Table 1), indicating that the balance shifting towards a proinflammatory effect in CPs might result in a higher incidence of CDI. For single inflammatory markers, ROC curve analysis showed that NLR in the total CPs and the > 18 yrs age group, MLR in the ≤ 18 yrs age group and the transsphenoidal group, and dNLR in the transcranial group were the most valuable predictive markers for postoperative CDI occurrence (Table 6; Fig. 1), indicating that preoperative peripheral neutrophils and monocyte can also mediate the effect of the proinflammatory response on postoperative CDI. Meanwhile, for the paired combination of these four markers, the best predictive performance for CDI was proven in the application of preoperative NLR + dNLR in CPs regardless of age and surgical approach (Table 6; Fig. 1), suggesting that the combination of preoperative peripheral NLR + dNLR might be used as a promising potential biomarker for postoperative CDI prediction in CP patients.

In addition, a growing number of researchers have reported that the location, removal rate and tumor volume of CPs can affect the occurrence of postoperative CDI [35, 36]. However, we did not draw these conclusions in this study. One possible reason of this inconsistency may depend on definition of the extent of tumor resection. Although we defined GTR as more than 95% of tumor resected and defined STR as 80–95% of tumor resected in this study [17, 18], majority of the reported investigations were stood on another standard as GTR as 100% and STR as more than 90% [37–40]. This may reflect the inconsistency between our study and previously reported statistical results. In addition, for tumor locations, QST classification was used. Previous studies have shown that patients with T-type CPs are more likely to have postoperative sodium metabolism disorder and hypothalamic-pituitary dysfunction [16]. In this study, only 23 of the 208 patients with CPs had T-type CPs, 17 of the 208 patients with CPs accept STR of the tumor. The small sample size may be the reason why this study did not reach the above conclusions, and further studies with larger sample sizes are needed in the future. Meanwhile, previous studies have suggested that the incidence of postoperative CDI in CP patients is not determined by a single factor, but by a combination of various factors such as GTR/STR removal rate, tumor location, tumor volume, surgical approach, etc., among which whether the pituitary stalk is preserved is particularly important [41–43]. The lack of further distinction between whether the tumor invaded the pituitary stalk and whether the pituitary stalk was preserved by surgery may be another reason why we were unable to reach the above conclusions. Of note, although there was no statistical significance, the location, removal rate and tumor volume of CPs had a tendency to affect postoperative CDI in this study (Table 1). Furthermore, although there was no significant difference, this study found that preoperative PSR might have an effect on preoperative inflammatory markers in patients with CPs, but this effect did not interfere with postoperative CDI (Table 1 and Supplementary Table 1). Due to the fact that the dose of corticosteroids could not be extracted from the patient’s medical records, the effect of cortisone dose on preoperative inflammatory markers and postoperative CDI could not be determined. Follow-up studies are needed to further prove whether the use of PSR will affect the stability of the prediction model constructed in this study.

There are still some limitations of this retrospective study. (1) Our study only collected data from a relatively small proportion of CP patients in a single clinical center. Therefore, multicenter studies and larger numbers of patients are needed to verify our preliminary results; (2) the time interval between CPs onset and blood collection are different in each patient, which might have caused bias in data collection due to the differences in the inflammatory response at different stages of disease.

## Conclusion

In this study, we observed that preoperative peripheral inflammatory markers, especially WBC, lymphocytes and NLR + dNLR, were promising predictors of postoperative CDI occurrence in CPs. This method of calculating preoperative circulation inflammatory markers can more accurately predict postoperative CDI and provide guidance for perioperative fluid management in CP patients.

### Electronic supplementary material

Below is the link to the electronic supplementary material.

Supplementary Material 1

## Data Availability

The datasets generated during and/or analyzed during the current study are available from the corresponding author on reasonable request.

## Abbreviations

AUC: Area under the curve
MRI: Magnetic resonance images
CDI: Central diabetes insipidus
NLR: Neutrophil-to-lymphocyte ratio
CNS: Central nervous system
PLR: Platelet-to-lymphocyte ratio
CP: Craniopharyngioma
PLT: Platelet
CSF: Cerebrospinal fluid
PSR: Preoperative steroid replacement
dNLR: Derived-NLR
ROC: Receiver operator characteristic
GTR: Gross total resection
STR: Subtotal resection
IQR: Interquartile range
WBC: White blood cells
MLR: Monocyte-to-lymphocyte ratio
