# Supplementary material for: Preoperative peripheral inflammatory markers are predictors of postoperative central diabetes insipidus in craniopharyngioma patients: a retrospective study
Source: BMC Cancer. 2024 May 8;24:572. doi: 10.1186/s12885-024-12324-4 (PMC11080258; doi:10.1186/s12885-024-12324-4)
Supplement: Supplementary file 1 — Supplementary Material 1 [file 12885_2024_12324_MOESM1_ESM.pdf]

Additional Table 1 Clinical analysis of hypopituitarism with PSR and inflammatory markers in craniopharyngioma patients

Inflammatory marker	Hypopituitarism with PSR	None	Z	*P*
WBC ($\times10^9$/L)	6.92 ± 2.32	7.58 ± 2.64	-1.024	0.306
Neutrophil ($\times10^9$/L)	3.50 ± 1.55	4.50 ± 2.27	-1.895	0.058
Lymphocyte($\times10^9$/L)	1.96 ± 0.44	1.96 ± 0.79	-0.553	0.580
Monocyte ($\times10^9$/L)	0.39 ± 0.11	0.47 ± 0.20	-1.820	0.069
PLT ($\times10^9$/L)	222.00 ± 46.71	226.25 ± 78.20	-0.318	0.751
NLR	1.81 ± 0.69	2.81 ± 2.20	-1.627	0.104
MLR	0.20 ± 0.06	0.27 ± 0.17	-1.840	0.066
dNLR	1.81 ± 1.07	1.77 ± 1.18	-0.275	0.784
PLR	118.37 ± 37.08	131.71 ± 79.85	-0.127	0.899

PSR: preoperative steroid replacement; WBC: white blood cells; PLT: platelet; NLR: neutrophil-to-lymphocyte ratio; MLR: monocyte-to-lymphocyte ratio; dNLR: derived NLR; PLR: platelet-to-lymphocyte ratio.
